# Neuro-Behçet With a Thalamic Lesion: A Case Report

**DOI:** 10.7759/cureus.45925

**Published:** 2023-09-25

**Authors:** Steven-Andrés Piña-Ballantyne, Luis-Angel Tirado-García, Martha-Lilia Tena-Suck, Ana-Laura Calderón-Garcidueñas

**Affiliations:** 1 Neuropathology, Instituto Nacional de Neurología y Neurocirugía Manuel Velasco Suárez, Mexico City, MEX; 2 Neuropathology, Instituto Nacional de Neurologia y Neurocirugía Manuel Velasco Suárez, Mexico City, MEX

**Keywords:** necrotizing vasculitis, behçet disease, neuro-behçet, perivascular infiltration, parenchymal, vasculitis

## Abstract

Behçet's disease (BD) is an autoimmune disease characterized by multisystemic variable-vessel vasculitis and oral, genital, and intestinal ulcers. Neurological involvement or “Neuro-Behçet" (NB) manifests due to parenchymal inflammation. We present the case of a 21-year-old male with a five-year-old history of intermittent chronic oral and genital ulcers who presented with headache, right hemiparesis, progressive loss of visual acuity, and a thalamic tumor-like lesion on magnetic resonance imaging (MRI). A brain biopsy showed multiple perivascular infarcts associated with vasculitis affecting arterioles, venules, and capillaries.

## Introduction

Behçet's disease (BD) or the “Silk Route disease” [1] is a rare multisystemic variable vessel vasculitis involving several systems [2]. The prevalence ranges from less than one per 1,000,000 cases in northern and central Europe to 2.5-20 per 1,000,000 cases in the western Mediterranean and eastern countries [3]. The male-female ratio in eastern countries is from 0.63-1.4 to 1 [3]. Symptoms manifest between the second and fourth decades of life, with an average onset between 20 and 35 years; however, it can occasionally occur in children [4]. The exact prevalence of pediatric BD is unknown, and some authors have reported that 4-26% of patients have pediatric onset [5].

Clinical symptoms include oral aphthae described in 98% of the cases, either single or multiple, in the tongue, pharynx, and labial and oral mucous membranes. Genital aphthae on the scrotum, penis, or urethra in men and on the vulva and vagina in women occur in 60-65% of cases. Eyes are generally associated with bilateral and severe affectation [6]; Behçet's uveitis (BU) presents in 50-90% of patients after at least 2-3 years of onset, however, BU may be the initial symptom in 10-20%. Furthermore, 16-25% of cases progress to blindness [7]. Skin involvement affects 38-99% of patients, and papulopustular and acne-like lesions are common [6]. Episodes of exacerbation and remission are frequent in this disease. The duration of the episodes ranges from a few days to a few weeks. They usually end in complete remission; however, sequelae can also occur [8].

Vascular involvement affects arterial and venous vessels with superficial thrombophlebitis, deep vein thrombosis, dural sinus thrombosis, occlusion, or aneurysm formation [4]. The disease course is more severe in young patients with the involvement of major organs [6].

BD diagnosis could be clinical or in support of imaging studies, according to the International Criteria for Behçet’s Disease (ICBD): oral aphthosis and genital aphthosis are each assigned 2 points, while skin lesions, central nervous system involvement, and vascular manifestations are assigned 1 point each. The pathergy test, when used, was assigned 1 point. Patients who scored ≥4 points were classified as having BD [9].

The pathogenesis remains unknown, although the association with HLA-B5 is seen in 40-65% of patients; in fact, HLA-B51 is the strongest genetic factor, even if it accounts for less than 20% of cases. Variations in the HLA-B51 allele explain its higher prevalence in eastern countries. Infectious diseases such as herpes viruses may play a pivotal role in pathogenesis [10].

Vasculitis involves both the arterial and venous systems, with a predominance in the venous system compared to other types of systemic vasculitis. It is possible to observe widespread vasculitis of arteries and venules of any size, involving nearly every system and organ [6]. The vessel walls undergo different changes due to infiltration by the surrounding inflammatory cells. In early lesions, neutrophil predominance is expected, and in chronic lesions, lymphocytes become more predominant [11].

We present the case of a 21-year-old male with a history of intermittent chronic oral and genital ulcers who presented with headache, right hemiparesis, and progressive loss of visual acuity. Magnetic resonance imaging (MRI) showed a thalamic lesion, and cerebrospinal fluid (CSF) tests were negative for neoplasia but positive for a mixed inflammatory process with mononuclear and polymorphonuclear cells. A brain biopsy was performed.

## Case presentation

A 21-year-old male with a two-month evolution condition characterized by progressive, bilateral loss in visual acuity and headache accompanied by sonophobia attended a neurological assessment. A week before admission, weakness in the right arm and leg, and an inability to hold objects; he also referred to dragging his leg while walking. At admission, somnolence, inattention, and disorientation were observed. He did not cooperate with the cognitive tests.

Previously, at age 16, he started experiencing painful intermittent oral and genital ulcers that spontaneously remitted. At that age, he required a blood transfusion due to iron deficiency anemia. Three months before visiting the hospital, he presented with multiple oral ulcers that spontaneously remitted; however, the patient reported this history retrospectively after a brain biopsy.

**Figure 1:**
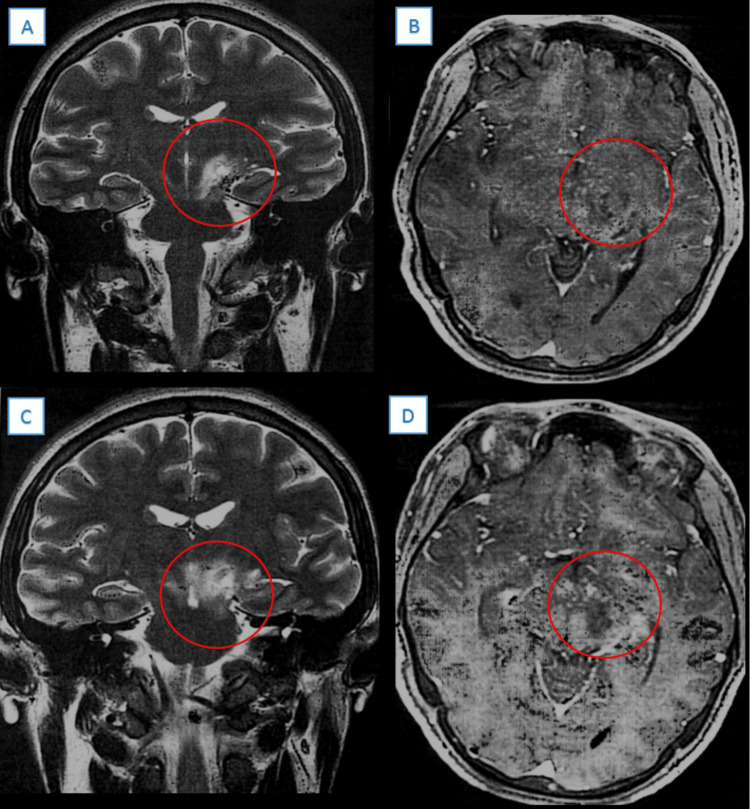
Cranial magnetic resonance imaging: (A) Coronal, T2; (B) Axial, T1; (C) Coronal, T2; (D) Axial, T1 A thalamic lesion (red circle), isointense on T1, and hyperintense on T2 with borders extending caudally toward the midbrain was observed.

A simple computed tomography (CT) showed hypodensities in the basal ganglia and left thalamus that extended to the mesencephalon. MRI showed a heterogeneous image of hypointense in T1 and hyperintense in T2 in the left basal ganglia and thalamus, extending caudally to the mesencephalon (Figure 1).

**Figure 2:**
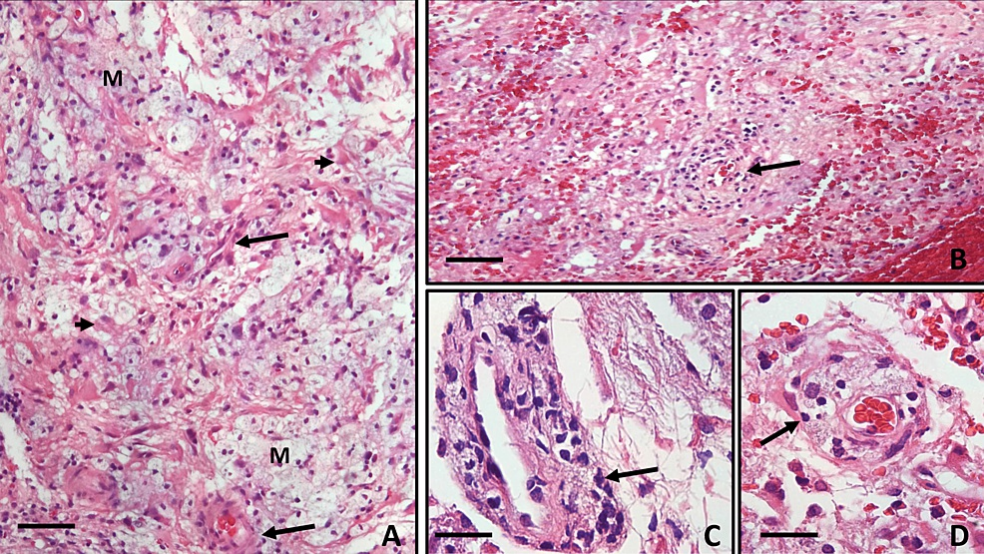
Sections of thalamic lesion, stained with hematoxylin and eosin. (A) Around small parenchymal vessels capillaries, venules, and arterioles (two large arrows) that showed variable degrees of damage, collarettes of foamy macrophages (M) were observed, in relation to infarcts, which tended to converge and were delimited by intense astrocytic reaction (two short arrows). (B-D) The vessels that were still feasible to identify showed thickening and edema of the wall and inflammatory infiltration with polymorphonuclear cells (black arrows) and some mature lymphocytes. (A) 50X (scale bar: 200 µm); (B) 100X (scale bar: 100 µm); (C-D) 400X (scale bar: 50 µm).

Basic laboratories, blood chemistry, liver profile, coagulation times, serum electrolytes, C-reactive protein (<0.30 mg/l), and general urinalysis were normal. Complete blood count showed leukocytosis (>12,000/mm^3^) with neutrophilia (>7,000/mm^3^), with no other alterations. An elevated erythrocyte sedimentation rate (42 mm/h) was detected. These studies were normal or negative: urine culture, blood culture (peripheral and central blood), HIV test, liver virus (hepatitis virus B and C), venereal disease research laboratory (VDRL) (syphilis), TORCH panel, enteroviruses, and Epstein-Barr virus. Three different CSF analyses showed non-specific inflammatory processes and were negative for malignancy (xanthochromic appearance; glucose 46-60; proteins 28-52; cells 24-38; 4-6 lymphocytes per field; 1-3 erythrocytes per field; 2-4 polymorphonuclear type cells per field; 1-3 macrophages per field). Given the fact that all the studies performed were negative, a biopsy of the thalamic lesion was performed. The patient had not received prior treatment with steroids or other immunosuppressants. Biopsy showed remanent blood vessels, both arterial and venous, with a polymorphonuclear inflammatory infiltrate with wall thickening and edema, and multiple infarcts (Figure 2).

**Figure 3:**
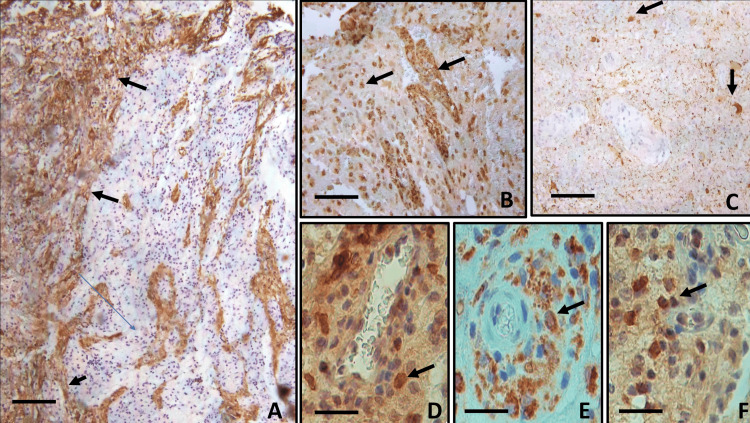
Immunohistochemistry (A) GFAP staining showed gliosis peripheral to macrophage collarettes and evidenced reactive astrocytes (three black arrows); (B) Perivascular collars are positive for CD68, as are stray cells in necrotic tissue (two arrows); (C) Synaptophysin showed some remaining neurons peripheral to the infarct areas (two black arrows); (D) In addition to polymorphonuclear cells, the presence of Th17 helper lymphocytes was evidenced in the vascular wall (black arrow); (E) HLA-DR+ activated macrophages (black arrow); and (F) cytotoxic lymphocytes (black arrow) in the stroma were identified. (A) 50X (scale bar: 200 µm); (B) 100X (scale bar: 100 µm); (C) 50X (scale bar: 200 µm); (D-F) 400X (scale bar: 50 µm). GFAP: glial fibrillary acidic protein, HLA-DR: human leukocyte antigen - DR isotype

The immunohistochemical study (Table 1) showed that the collarette cells were positive for CD68, confirming their histiocytic lineage and that they also expressed HLA-DR (activation). The inflammatory infiltrate was mixed with CD4 lymphocytes (Th lymphocytes), including Th17 (proinflammatory) lymphocytes and CD8 (cytotoxic lymphocytes). Staining for the mutated isocitrate dehydrogenase enzyme (IDH1/2) and the proliferation antigen Ki-67 were negative. PAS and Ziehl-Neelsen stains did not show fungus or tuberculosis bacilli (Figure 3).

**Table 1:** Antibodies used in the immunohistochemistry panel GFAP: glial fibrillary acidic protein; IDH: isocitrate dehydrogenase; HLA-DR: human leukocyte antigen - DR isotype; CA: California; TX: Texas

Antibody	Cellular Localization	Description	Dilution	Company	Location	Catalog Number
GFAP	Cytoplasm	1.0 ml	1:100	Biogenex	Fremont, CA	MU020-UC
Synaptophysin	Cytoplasm	1.0 ml	1:100	Diagnostic Biosystems	Pleasanton, CA	Mob399
CD68	Cytoplasm	1.0 ml	1:25	Biogenex	Fremont, CA	MU416-UC
CD4	Cell membrane and cytoplasm	1.0 ml	1:10	Biogenex	Fremont, CA	MU421-UC
CD8	Cell membrane and cytoplasm	1.0 ml	1:15	Biogenex	Fremont, CA	MU261-UC
IL-17 (Th17)	Cytoplasm	1.0 ml	1:250	Santa Cruz Biotechnology	Dallas, TX	sc7927
IDH1/2	Cytoplasm	1.0 ml	1:250	Santa Cruz Biotechnology	Dallas, TX	sc373816
KI67	Nucleus	1.0 ml	1:10	Biogenex	Fremont, CA	MU410-UC
HLA-DR	Cell membrane	1.0 ml	1:50	Diagnostic Biosystems	Pleasanton, CA	Mob069R

BD was diagnosed by meeting 6 points of the ICBD [9]: oral (2 points) and genital (2 points) aphthae, vascular manifestations (1 point), and neurological manifestations (1 point). The histological diagnosis was compatible with BD.

**Figure 4:**
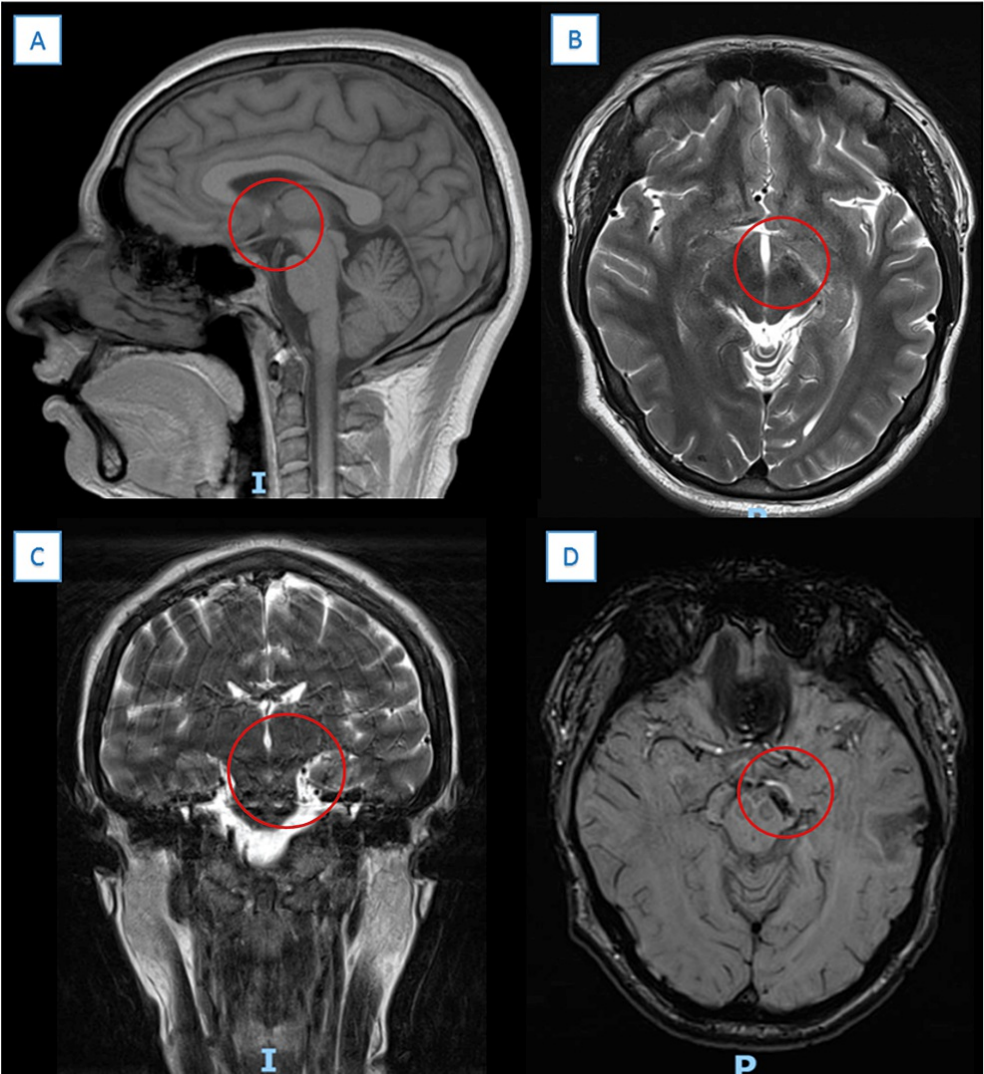
Cranial magnetic resonance imaging (MRI) after one year of surveillance: (A) Sagittal, T1; (B) Axial, T2; (C) Coronal, T2; (D) Axial, susceptibility-weighted imaging (SWI). Encephalomalacia area affecting the most ventral portion of (A) the globus pallidus (red circle), (B, D) the posterior arm of the left internal capsule (red circle), and (C) the left cerebral peduncle. The left cerebral peduncle and ipsilateral pyramid were reduced in size. No new lesions were observed, only chronic post-surgical changes.

During his postsurgical recovery, the patient developed a painful ulcer in the scrotum with well-defined edges and a purulent appearance. Intravenous ceftriaxone (1g/day for five days), boluses of intravenous (IV) methylprednisolone (1g/kg/day for five days), and one bolus of cyclophosphamide B (1 g per square meter of body surface) were administered. Due to clinical improvement, the patient was discharged with outpatient treatment, ceftriaxone (1g/day orally for nine days), prednisone (1 mg/kg/day), and monthly boluses of cyclophosphamide. After one year of follow-up with no complications or recurrences, a normal neurological examination, and a follow-up MRI were performed. Chronic postsurgical changes were identified and no new lesions were observed (Figure 4).

## Discussion

BD is a rare multisystemic variable vessel vasculitis that involves several systems and mainly causes ulcers in the oral, genital, and intestinal mucosa [2]. The least common complications include arthritis and gastrointestinal, cardiovascular, and neurological involvement [12]. The term “Neuro-Behçet” (NB) was credited to the Italian ophthalmologists Cavara and D’Ermo in 1954 [13]. NB occurs in 5-10% of patients, commonly in men, with a male-to-female ratio of 4:1 [4]. The central nervous system (CNS) is more affected than the peripheral nervous system [4,6]. It usually appears within five years after the onset of the disease [4]. There are two primary phenotypes: parenchymal and non-parenchymal. The more prevalent parenchymal involvement primarily targets the brainstem and basal ganglia; however, instances of spinal cord and hemispheric lesions have also been documented. In contrast, non-parenchymal engagement encompasses dural sinus thrombosis, arterial occlusion, and aneurysms [4]. These lesions frequently stem from vasculitis-induced thrombosis [14].

The predominant immunological profile includes the proinflammatory activity of Th1, activation of Th1 and Th2 cytokines, and neutrophil hyperactivity [4,12]. Neutrophils show exaggerated chemotaxis when exposed to plasma from patients with BS, and release neutrophil extracellular traps (NETs), which are prothrombotic web-like structures, and excessive amounts of reactive oxygen species (ROS) that promote fibrinogen oxidation and thrombus formation [4]. Activation markers such as CD64, which is a high-affinity receptor that binds to the Fc region of immunoglobulin G (IgG), are increased in patients with active BD. Other studies have shown that CD56 natural killer (NK) cells are depleted in peripheral blood, which correlates with disease activity [12]. Increased IL-8, tumor necrosis factor (TNF-α), and IL-1 production by lymphomononuclear and endothelial cells are other findings in BD [15]. In fact, numerous Th17 cells were observed in the vessel walls of our patient.

The diagnosis of NB is not always easy, especially if, at the time of diagnosis, the history of oral and genital ulcers is not provided, since patients often do not associate ulcers with neurological problems and forget previous episodes.

In the context of a young patient with a history of oral and genital ulcers, presenting with a parenchymal brain or cerebral vascular lesion, one is compelled to think of NB and to rule out other possible etiologies. This case is exceptional because it was necessary to obtain a biopsy to rule out differential diagnoses, including infections and neoplasia. As the most frequently affected sites include the brainstem, especially the meso-diencephalic junction, cerebellar peduncles, and basal ganglia [16], taking a biopsy has technical difficulties. On the other hand, findings depend on the stage of the disease and the degree of inflammation. Therefore, there are few reports on brain biopsies in patients with BD [17]. In the early stages, the main histopathological feature of CNS lesions is the perivascular infiltration of mononuclear and polymorphonuclear leukocytes and eosinophilic cells [18]. Other cases of meningeal infiltration, axonal loss, and gliosis have been reported in the late stages [19]. Other uncommon findings include multifocal necrosis, glial proliferation, and demyelination [19]. Hirohata reported histopathological changes in the CNS of three autopsy cases: cuffing of mononuclear leukocytes infiltrating around small vessels that consisted of T lymphocytes and monocytes/macrophages. Immunohistochemistry demonstrated that these cells were CD68-positive macrophages, CD45RO-positive T lymphocytes, and a few CD20-positive B lymphocytes [19]. Our histopathological examination demonstrated the presence of multiple confluent perivascular infarcts. The remaining vessels showed vasculitis with marked edema of the wall and endothelial cells and a polymorphonuclear infiltrate in the vascular wall, in addition to lymphocytic infiltrates, especially Th17 cells.

Differential diagnoses included multiple sclerosis, systemic lupus erythematosus, Wegener granulomatosis, primary antiphospholipid antibody syndrome, primary angiitis of the CNS, brain tumors, tuberculous meningitis, sarcoidosis, Lyme disease, Brucella meningitis, and other causes of chronic meningitis. Similar to many chronic relapsing inflammatory disorders, treatment options for NB include relapse, long-term attack prevention, and symptomatic treatment [4].

Actually, the patient is neurologically intact, although he receives psychological support for anxiety and lack of motivation.

## Conclusions

We present the case of a 21-year-old male with BD and a complex presentation with a thalamic lesion and a CSF with a non-specific inflammatory process. A biopsy was performed that showed a diffuse, mixed inflammatory process with vasculitis, parenchymal infarct, and reactive gliosis. Vasculitis with infiltration of the vascular wall with polymorphonuclears and infarcts represents a challenge to pathologists when observed without a clinical background. By integrating all the findings, a diagnosis of NB was made. To date, biopsy remains the last line of study for diagnosis, and very few histopathological articles on NB have been published.
